# Supplementary material for: Pretreatment with Citrus reticulata ‘Chachi’ Polysaccharide Alleviates Alcohol-Induced Gastric Ulcer by Inhibiting NLRP3/ASC/Caspase-1 and Nrf2/HO-1 Signaling Pathways
Source: Nutrients. 2025 Jun 20;17(13):2062. doi: 10.3390/nu17132062 (PMC12250903; doi:10.3390/nu17132062)
Supplement: Supplementary file 1 [file nutrients-17-02062-s001.zip › nutrients-3670730-supplementary.pdf]

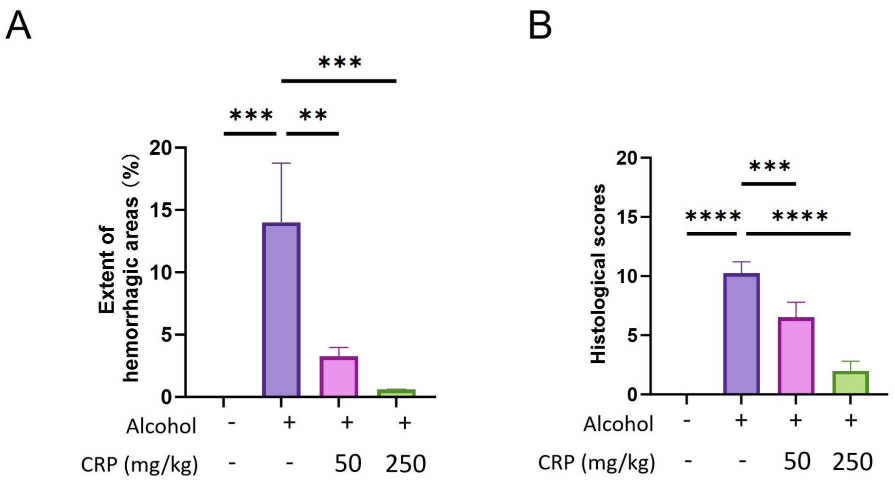

Figure S1. Effect of CRP on body weight and gastric damage in alcohol-induced GU mice. (A) Extent of hemorrhagic areas. (B) H&E histological scores. Data are presented as means ± SD (n = 8). *P < 0.05, **P < 0.01, ***P < 0.001, ****P < 0.0001 versus the alcohol-induced group

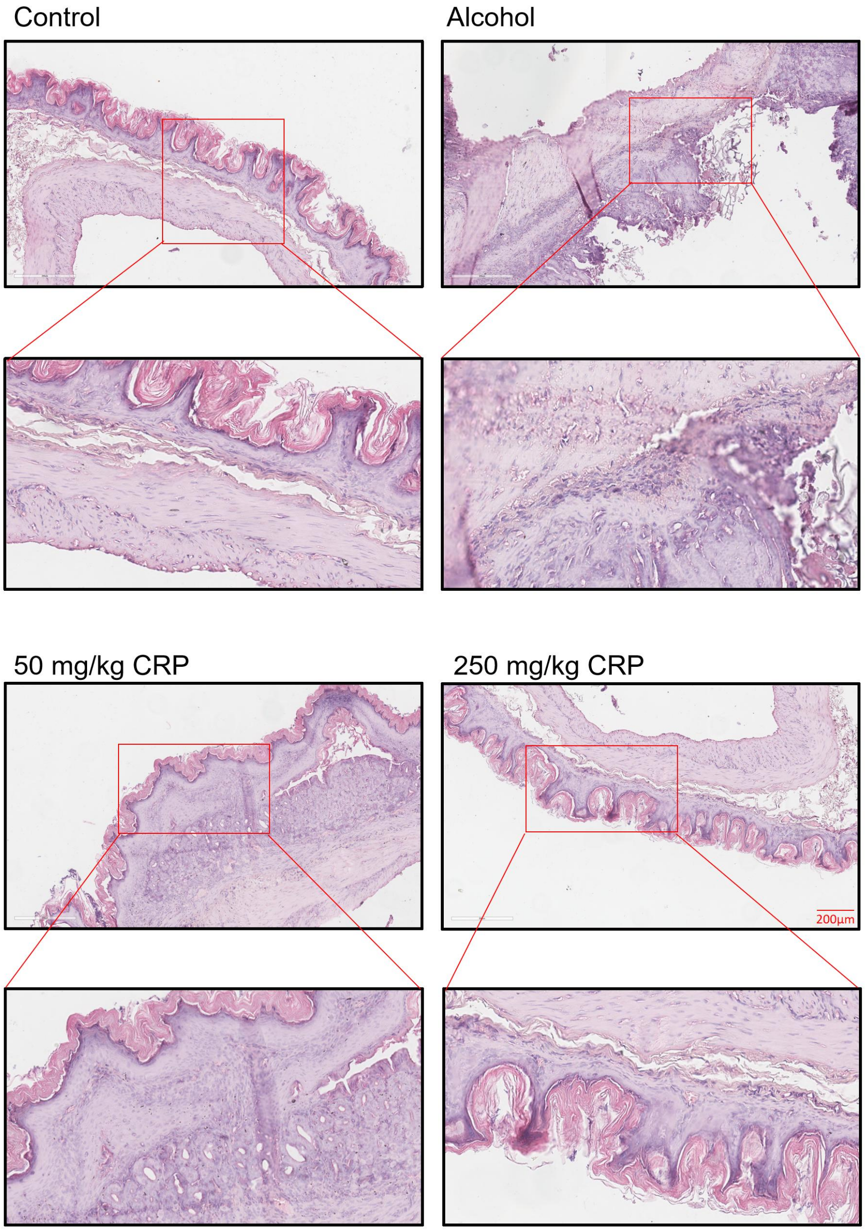

Figure S2. Local magnification of the HE staining of the gastric tissues.

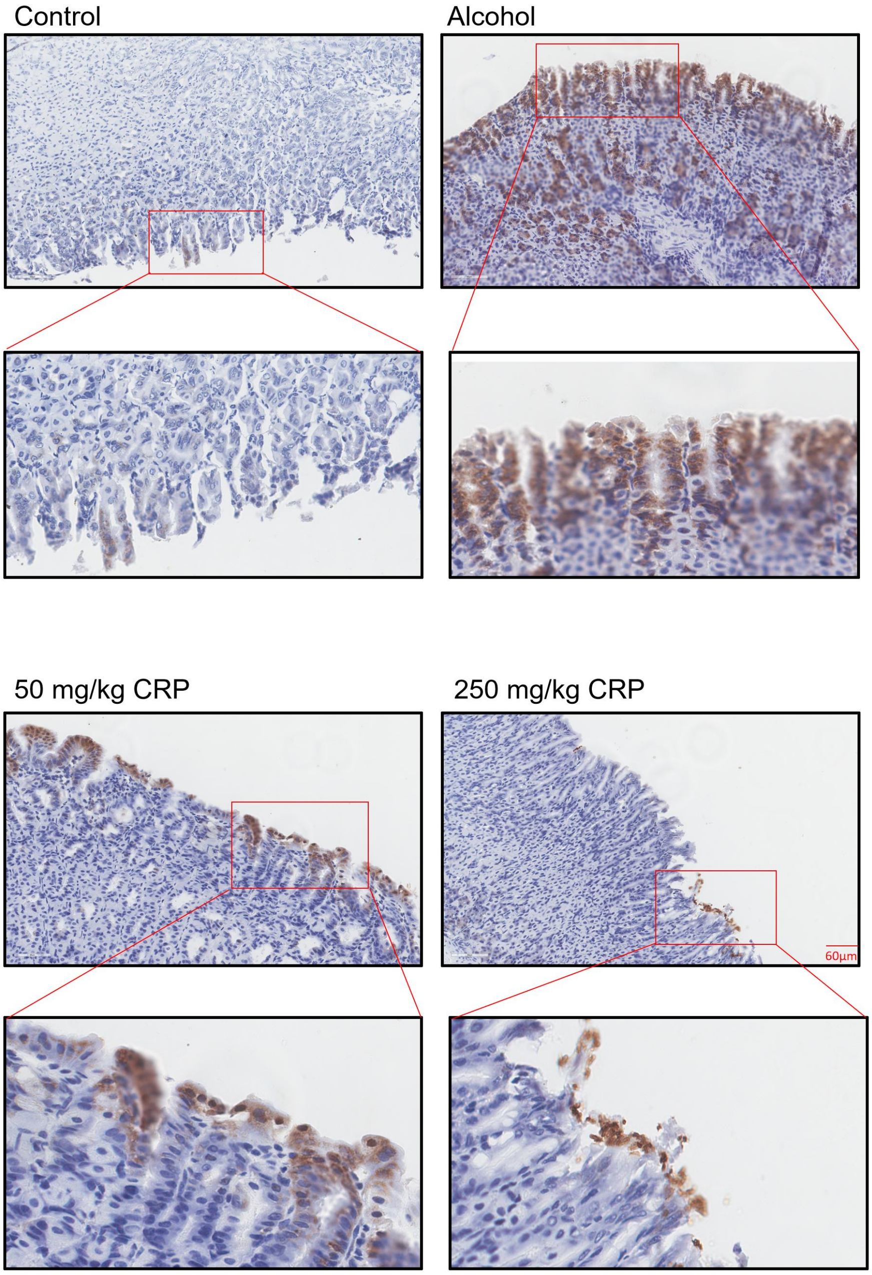

Figure S3. Local magnification of the IHC staining for iNOS.

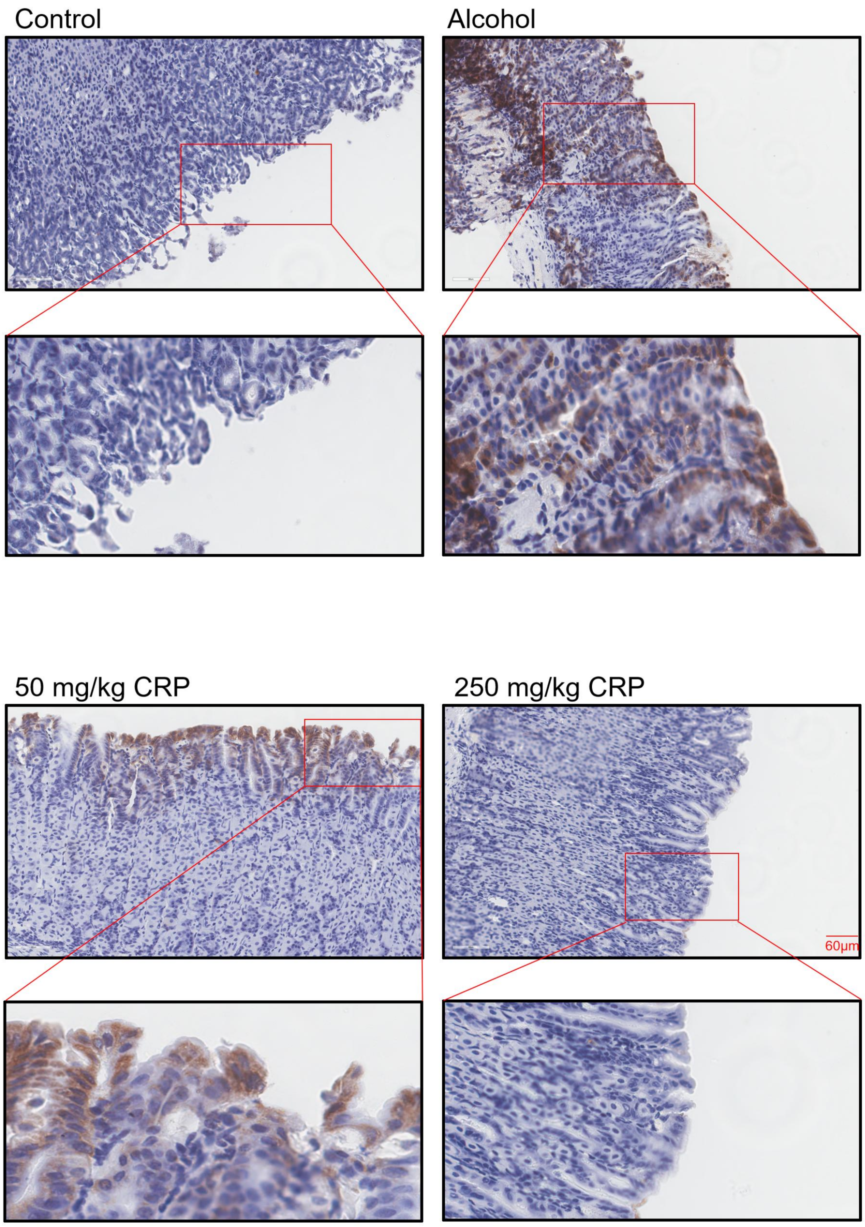

Figure S4. Local magnification of the IHC staining for NLRP3.

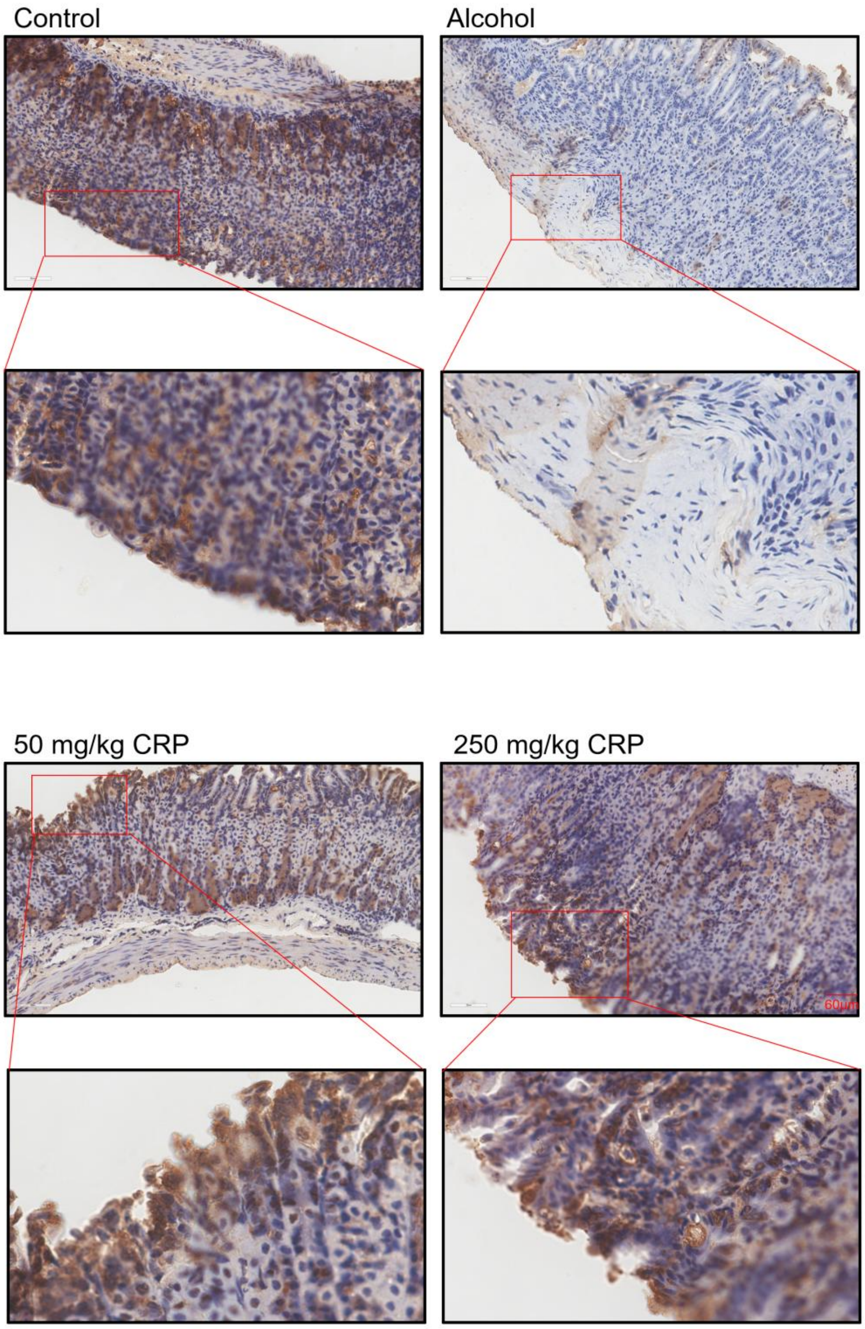

Figure S5. Local magnification of the IHC staining for Nrf2.

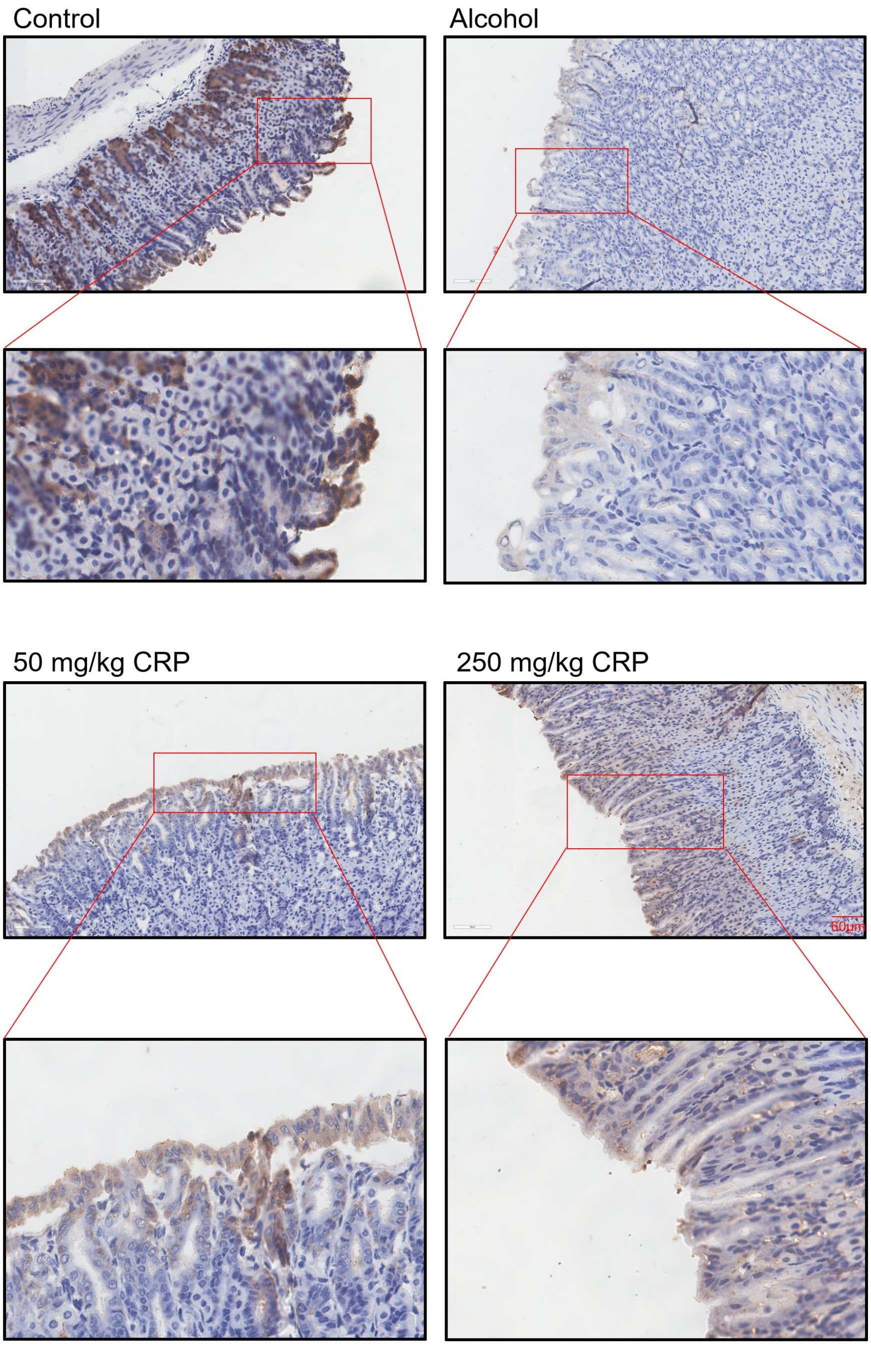

Figure S6. Local magnification of the IHC staining for ZO-1.

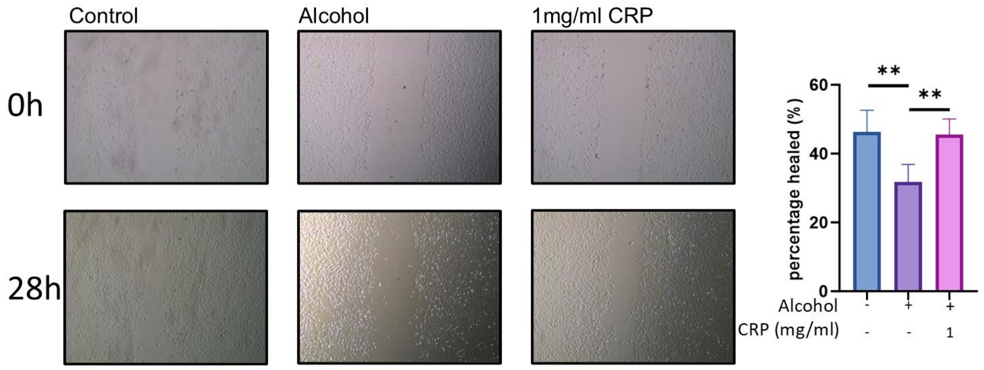

Figure S7. effect of CRP on wound healing capacity. Data are presented as means ± SD (n = 8). *P < 0.05, **P < 0.01 versus the alcohol-induced group.

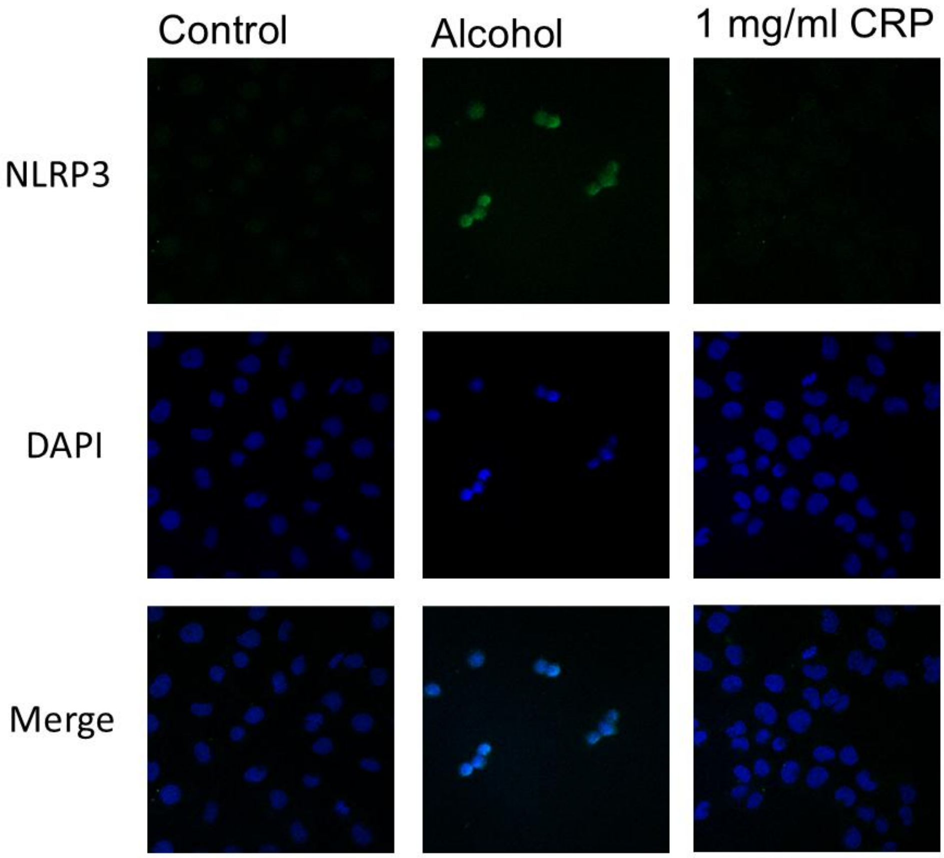

Figure S8. Immunofluorescence staining of NLRP3 in GES-1 cells.

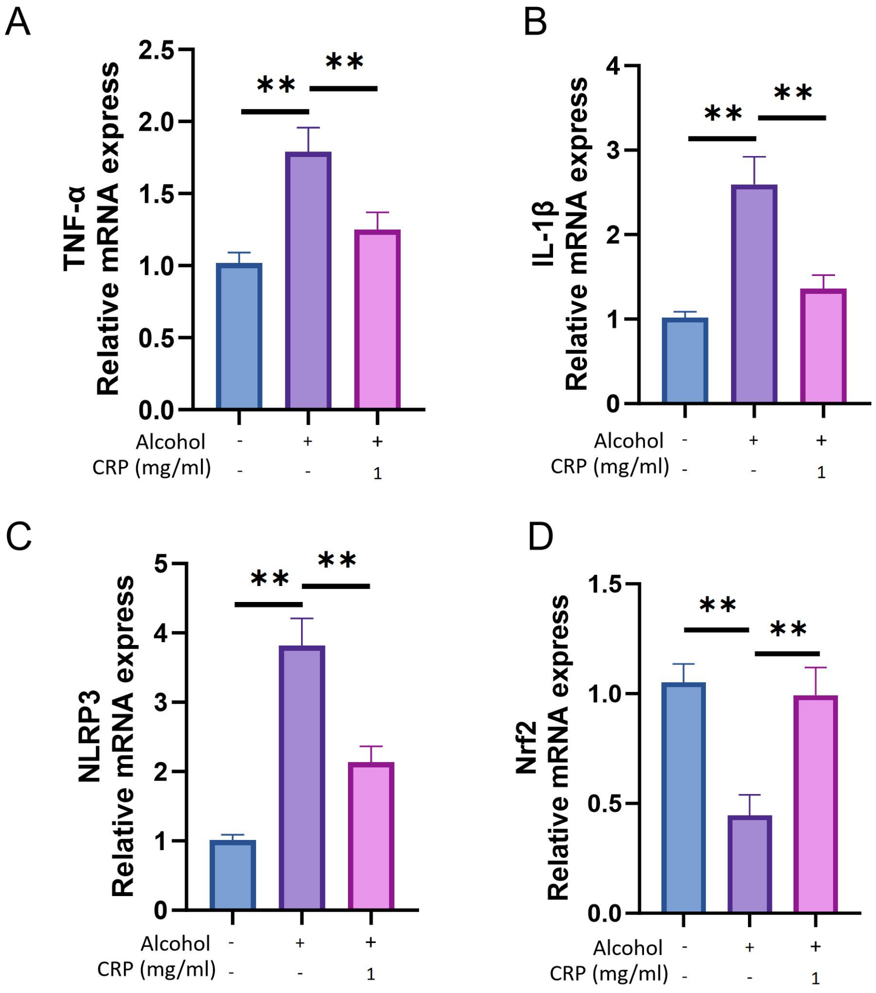

Figure S9. Effects of CRP on the mRNA expression of TNF-α (A), IL-1β (B), NLRP3 (C) and Nrf2 (D) in the GES-1 cells. Data are presented as means ± SD (n =5). *P < 0.05, **P < 0.01 compared to the alcohol-induced group.

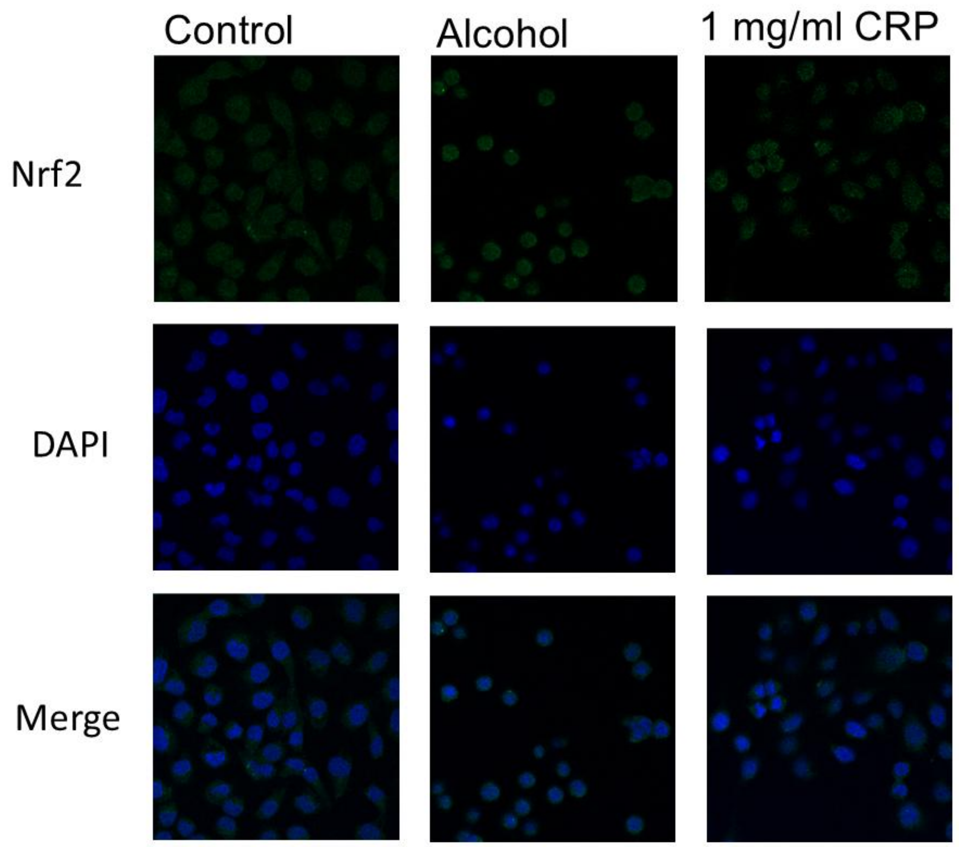

Figure S10. Immunofluorescence staining of Nrf2 in GES-1 cells.

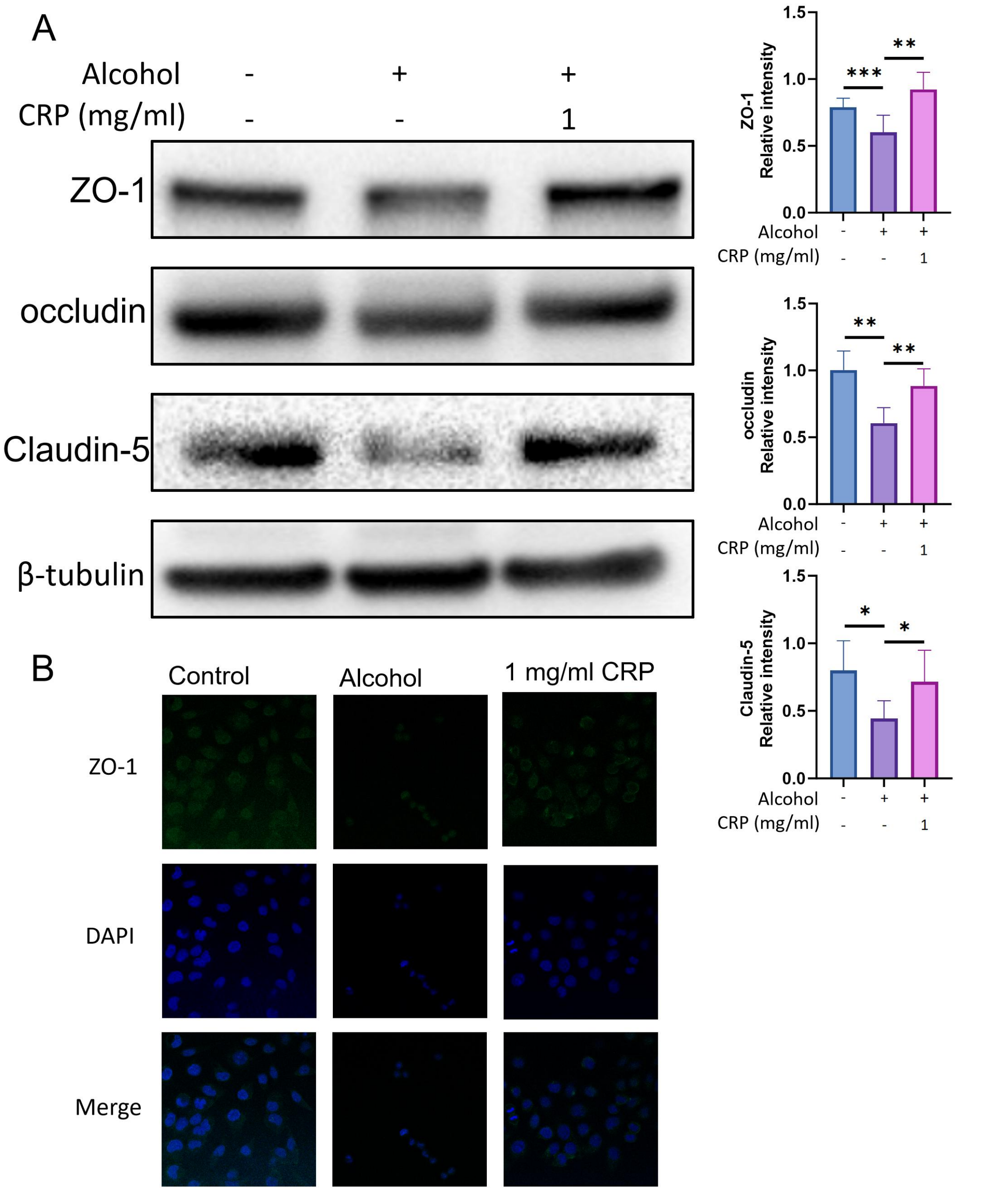

Figure S11. Effect of CRP on alcohol-induced disruption of tight junction proteins in gastric epithelial cells. (A) Western blot analysis of tight junction proteins ZO-1, occludin, and claudin-5 in GES-1 cells. β-tubulin was used as a loading control. Data are presented as means ± SD, n = 8. *P < 0.05, **P < 0.01, ***P < 0.001, compared to the alcohol-induced group. (B) Immunofluorescence staining of ZO-1 in GES-1 cells.

Table S1. H&E Staining Histological Score Table

Grading Standards	Details	Score
1. Mucosal structural integrity	Mucosal structure is intact, there is no damage, and glands are arranged neatly.	0
	Local superficial epithelial cells shed or slightly eroded, and the gland structure was slightly disordered.	1
	Moderate erosion or ulcer, involving 1/2 of mucous membrane, destruction of gland structure.	2
	Deep ulcer or full-thickness mucosal loss involving submucosa or muscularis.	3
2. Inflammatory cell infiltration	No inflammatory cell infiltration	0
	A few inflammatory cells (such as neutrophils and lymphocytes) are scattered.	1
	Moderate inflammatory cell infiltration and local aggregation	2
	Extensive inflammatory cell infiltration, forming obvious inflammatory lesions.	3
3. Edema and congestion	No edema or congestion	0
	Mild submucosal edema or local congestion	1
	Moderate edema with extensive congestion, mucosal layer separation	2
	Severe edema accompanied by vasodilation, and the tissue gap was significantly widened.	3
4. Hemorrhage and necrosis	No bleeding or necrosis.	0
	Punctate bleeding or a small number of necrotic cells	1
	Flaky hemorrhage or local necrosis focus	2
	Extensive bleeding or extensive necrosis, involving deep tissue.	3

Table S2. Primer sequence.

Gene	Forward	Reverse
TNF-α	GCCACCACGCTCTTCTGT CTAC	GGGTCTGGGCCATAGAACTGA T
IL-1β	CACCTCTCAAGCAGAGC ACAG	GGGTTCCATGGTGAAGTCAAC
NLRP3	ATTACCCGCCCGAGAAA GG	CATGAGTGTGGCTAGATCCAA G
Nrf2	AACCAGTGGATCTGCCA ACTACT C	CTGCGCCAAAAGCTGCAT
GAPDH	GCACCGTCAAGGCTGAG AAC	TGGTGAAGACGCCAGTGGA
